# Aurora Medical Association

**Published:** 1854-04

**Authors:** 


*Aurora Medical Association.*

At a meeting called for the purpose of forming a Medical Society, pursuant to an adjournment of a preliminary meeting held at Aurora, March 6th, Dr. O. A. Lovejoy was called to the chair and Dr. A. Hard was appointed Secretary.

The preliminary steps were then taken for the organization of a Society, to be known as the Aurora Medical Association, and Drs. Angell, Young, and Hard, were appointed a Committee to prepare a Constitution and By-laws, for its permanent organization.

On motion, the Meeting adjourned to meet at 7 P. M.

Meeting called to order by the President. Dr. Angell, Chairman of the the Committee, reported that they had performed the duty assigned them; which report was accepted, and the Constitution and By-laws were, with some amendments, adopted.

The following permanent officers, were then elected for the current year, viz: Dr J. L. Brooks, President; Drs. L. H. Angell, and J. E. Carr, Vice-Presidents; Dr. O. A. Lovejoy, Secretary; P. A. Allaire, Corresponding Secretary; Dr. D. W. Young, Treasurer; Drs. D. W. Young, J. E. Carr, and A. Hard, Censors.

On motion, the Secretary was requested to publish in such Journals as he thought proper, an abstract of the proceedings of this meeting. When,

On motion, the Association adjourned to meet again the 2nd Monday in April. J. L. BROOKS, Pres't.

O. A. LOVEJOY, Secretary.

---

A regular meeting of the Association was held at Aurora on the 10th inst.

Dr. D. W. Young, was appointed delegate to the American Medical Society. Dr. J. E. Carr, Substitute.

Dr. P. A. Allaire was appointed delegate to the State Medical Society. Dr. A. Hard, Substitute.

Dr. Ira Dales presented his credentials, and was admitted as a member of the Association. Drs. C. Wheeler and D. Eastman were admitted as Honorary Members.

Various matters of professional interest, were talked of and discussed, after which the association adjourned to meet again on the first Monday in May. O. A. LOVEJOY, Sec'y.

Aurora, April 13th, 1854.

